# Evaluating a multi-component, community-based program to improve adherence and retention in care among adolescents living with HIV in Zimbabwe: study protocol for a cluster randomized controlled trial

**DOI:** 10.1186/s13063-017-2198-7

**Published:** 2017-10-20

**Authors:** Webster Mavhu, Nicola Willis, Juliet Mufuka, Collin Mangenah, Kudzanayi Mvududu, Sarah Bernays, Walter Mangezi, Tsitsi Apollo, Ricardo Araya, Helen A. Weiss, Frances M. Cowan

**Affiliations:** 1 Centre for Sexual Health and HIV/AIDS Research (CeSHHAR), 9 Monmouth Road, Avondale West, Harare, Zimbabwe; 2 Department of International Public Health, Liverpool School of Tropical Medicine, Liverpool, UK; 3 Africaid, Harare, Zimbabwe; 4 School of Public Health, University of Sydney, Sydney, NSW Australia; 5 Department of Psychiatry, University of Zimbabwe College of Health Sciences, Harare, Zimbabwe; 6 AIDS and TB Unit, Ministry of Health and Child Care, Harare, Zimbabwe; 7 Health Services and Population Research Department, King’s College London, London, UK; 8 MRC Tropical Epidemiology Group, London School of Hygiene and Tropical Medicine, London, UK

**Keywords:** HIV, Antiretroviral therapy, Adolescents, Adherence, Zimbabwe, Psychosocial, Community-based

## Abstract

**Background:**

World Health Organization (WHO) adolescent HIV-testing and treatment guidelines recommend community-based interventions to support antiretroviral therapy (ART) adherence and retention in care, while acknowledging that the evidence to support this recommendation is weak. This cluster randomized controlled trial aims to evaluate the effectiveness and cost-effectiveness of a psychosocial, community-based intervention on HIV-related and psychosocial outcomes.

**Methods/design:**

We are conducting the trial in two districts. Sixteen clinics were randomized to either enhanced ART-adherence support or standard of care. Eligible individuals (HIV-positive adolescents aged 13–19 years and eligible for ART) in both arms receive ART and adherence support provided by adult counselors and nursing staff. Adolescents in the intervention arm additionally attend a monthly support group, are allocated to a designated community adolescent treatment supporter, and followed up through a short message service (SMS) and calls plus home visits. The type and frequency of contact is determined by whether the adolescent is “stable” or in need of enhanced support. Stable adolescents receive a monthly home visit plus a weekly, individualized SMS. An additional home visit is conducted if participants miss a scheduled clinic appointment or support-group meeting. Participants in need of further, enhanced, support receive bi-weekly home visits, weekly phone calls and daily SMS. Caregivers of adolescents in the intervention arm attend a caregiver support group. Trial outcomes are assessed through a clinical, behavioral and psychological assessment conducted at baseline and after 48 and 96 weeks. The primary outcome is the proportion who have died or have virological failure (viral load ≥1000 copies/ml) at 96 weeks. Secondary outcomes include virological failure at 48 weeks, retention in care (proportion of missed visits) and psychosocial outcomes at both time points. Statistical analyses will be conducted and reported in line with CONSORT guidelines for cluster randomized trials, including a flowchart.

**Discussion:**

This study provides a unique opportunity to generate evidence of the impact of the on-going Zvandiri program, for adolescents living with HIV, on virological failure and psychosocial outcomes as delivered in a real-world setting. If found to reduce rates of treatment failure, this would strengthen support for further scale-up across Zimbabwe and likely the region more widely.

**Trial registration:**

Pan African Clinical Trial Registry database, registration number PACTR201609001767322 (the Zvandiri trial). Retrospectively registered on 5 September 2016.

## Background

In sub-Saharan Africa, the number of HIV-related deaths among adolescents (aged 10–19 years) tripled between 2000 and 2014 despite decreasing among all other age groups. HIV is now the leading cause of death among this age group [1, 2]. The increase in adolescent HIV-related deaths has been attributed to poor prioritization of adolescents in national HIV plans, inadequate provision of accessible and acceptable HIV-testing, counseling and treatment services for this age group, plus a lack of tailored support for adolescents to remain in care and adhere to taking antiretroviral therapy (ART) [3–5]. Adolescents in sub-Saharan Africa have poorer ART adherence than adults [6] and are at high risk of loss to follow-up from treatment and care [7]. This supports findings among adolescents with other chronic childhood illnesses (e.g., diabetes, asthma) when medication adherence frequently becomes worse as the child becomes more independent from parental control as they enter adolescence [8].

In 2013, the World Health Organization (WHO) released the first consolidated ART prevention and treatment guidelines which recommended innovative models of ART delivery (decentralization, integration and task-shifting) [9]. In response, Zimbabwe revised its national ART guidance, released in December 2013, to simplify recommended treatment regimens for children and adolescents, hence facilitating the decentralization of pediatric HIV care to primary healthcare facilities [10]. Decentralizing pediatric HIV care has resulted in improved access and coverage, but little is known about the capacity of primary care facilities to provide effective adherence support as well as support for the broader psychosocial issues that many of these children and adolescents face [4].

A key tenet of both the WHO and Zimbabwe’s treatment guidelines is that expanded access to ART needs to be accompanied by interventions to maximize retention in care and medication adherence. Poor retention and adherence to ART leads to treatment resistance and ultimately, to treatment failure [5, 11, 12]. International guidelines recommend at least 95% adherence to ART to minimize the risk of virological failure [13–15]. A systematic review of 17 studies relating to ART programs in children and adolescents from low- and middle-income countries (LMIC) found that non-adherence was associated with a child’s socioeconomic environment, stigma, caregiver characteristics, dynamics of child-caregiver interactions, disclosure of HIV status, regimen complexity, side effects, and cost of medication [16].

The 2013 and 2015 WHO adolescent HIV-testing and treatment guidelines recommend community-based interventions to support ART adherence and retention in care, while acknowledging that the evidence to support this recommendation is weak [4, 17]. Evidence-based strategies to provide support to the growing number of adolescents receiving ART from LMIC are urgently required. Preliminary evidence suggests that the Zvandiri (“As I am”) program (http://www.africaid-zvandiri.org/) has the potential to make a significant impact on the lives of adolescents living with HIV [18].

The Zvandiri program is a model of differentiated clinical service delivery for HIV-positive children and adolescents in Zimbabwe, has been cited as a “best practice” intervention by the WHO [4], USAID, Southern African Development Community [19], and has been recommended for adaptation and regional scale-up [19]. The program uses trained and mentored community adolescent treatment supporters (CATS) to deliver structured support groups and tailored community-based adherence support coupled with counseling for the broader psychosocial issues that these young people are facing (including coming to terms with their emerging sexuality in the context of a life-threatening, sexually transmissible infection). The CATS are aged 18–23 years, living with HIV, have been identified by the healthcare facility as committed, competent and motivated to support their peers as well as adherent to ART, having completed secondary schooling and having consent from their caregivers to enroll as CATS. The Zvandiri program also includes an intervention for caregivers which focuses on enhancing their knowledge, skills and confidence of HIV and treatment literacy as well as communication and parenting through a participatory training program.

In this paper we describe the protocol for a cluster randomized controlled trial (cRCT) to evaluate the effectiveness and cost-effectiveness of the Zvandiri program on HIV-related and psychosocial outcomes.

## Methods/design

### Objectives

The trial will provide evidence on whether enhancing community-based support for adolescents on ART through the Zvandiri program will, compared with standard care:

1. Reduce the proportion of adolescents with ART treatment failure (lack of viral suppression)
2. Improve retention in care
3. Reduce the severity of anxiety and depression
4. Reduce the proportion of adolescents reporting non-disclosure
5. Be cost-effective

### Hypotheses

The primary hypothesis is that the Zvandiri program will be more effective than standard care in reducing the proportion of adolescents who have died or who are failing treatment at 96 weeks (defined as a viral load ≥ 1000 copies/ml).

The secondary hypotheses are that the Zvandiri program (1) improves virological failure at 48 weeks, (2) improves retention in care, (3) reduces psychological distress, (4) increases HIV status disclosure to sexual partners and (5) decreases perceived stigma.

### Trial setting

The trial is being conducted in two districts (Bindura and Shamva) in Mashonaland Central Province. The districts were selected in consultation with the Ministry of Health and Child Care (MoHCC). At the time of selection, it was estimated that 24,871 adolescents were living with HIV in the province and that ART coverage among children and adolescents was the lowest in Zimbabwe (estimated to be 29%). Additionally, although the Zvandiri program was being scaled-up, it had not yet been initiated in these two districts. Altogether, there are 33 healthcare facilities in the two districts. Of these, 16 with at least 20 adolescents on ART (excluding private clinics and those not yet offering pediatric HIV treatment and care services) were selected to participate.

### Trial design

**Fig. 1:**
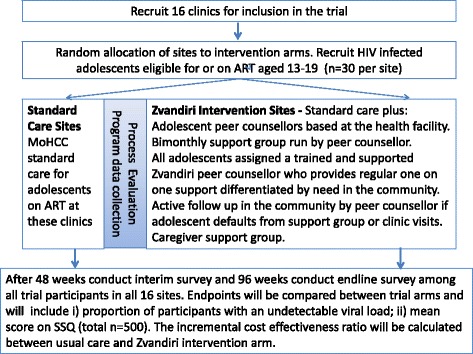
Trial overview

The trial is a cluster randomized controlled trial (cRCT). The clinic is the unit of randomization. Sixteen clinics were randomized to either enhanced ART-adherence support through the Zvandiri program or standard of HIV care in a 1:1 allocation (Fig. 1). Adolescents aged 13–19 years were recruited. Trial outcomes are assessed through a clinical, behavioral and psychological assessment conducted at baseline and after 48 and 96 weeks.

### Eligibility criteria

1. Inclusion criteria: HIV-positive adolescents, aged 13–19 years, eligible for ART (i.e., either starting or already on ART) and able to provide informed assent and their caregiver is able to provide informed consent (those aged 18 and 19 years do not need caregiver consent)
2. Exclusion criteria: too physically or psychologically unwell or unable to give informed consent

### Interventions

#### Control arm

All eligible adolescents attending clinics allocated to the standard-care arm receive ART and adherence support as set out in the prevailing MoHCC guidelines [20]. As per MoHCC guidelines, adherence support is provided by adult counselors and nursing staff. After ART initiation, adolescents are seen monthly, with cluster of differentiation 4 (CD4) monitoring at 6-monthly intervals. Prescription refills, pill counts and participant interviews are used to measure adherence. Although routine viral-load testing was adopted by MoHCC in 2010 and scale-up began in 2012 [21], the MoHCC is currently only able to provide targeted rather than universal viral-load testing.

#### Intervention arm

All eligible adolescents attending intervention clinics will receive MoHCC standard care. In addition, one to three (ideally at least one man, one woman) trained and supported CATS are at each clinic to provide adherence counseling and support to adolescents at their clinic visits as well as on-going individualized community-based support. Each CATS supports 6–10 adolescents in their geographical location irrespective of level of Zvandiri support. CATS were recruited from the clinic in partnership with the clinic staff, or from the local support group if a group was already in place.

The CATS receive 2 weeks of MoHCC-endorsed training to equip them with counseling skills and skills in community outreach plus knowledge specific to adolescent HIV (ART and adherence, disclosure, sexual and reproductive health, psychosocial support and mental health). The training combines both theoretical and practical components, including the “shadowing” of existing CATS in Harare to acquire practical experience of providing adherence support. Training includes learning how to identify “red flags” for ill-health, adherence challenges, psychosocial distress and common mental disorders (CMDs), plus basic skills in problem solving and cognitive behavioral therapy (CBT). Training is participatory and uses case studies. All CATS attend a weekly supervision meeting at the clinic with a designated nurse and provide each other with peer-to-peer support (through a WhatsApp group and via Skype from Harare) with oversight from a professional counselor. Trial participants are allocated to a designated CATS according to their residential area and followed up through the short message service (SMS), phone calls and home visits. The type and frequency of contact is determined following assessment of their individual situation (i.e., whether they are considered “stable” or in need of enhanced support):

1. “Stable” adolescents are those: (a) with a viral load < 1000 copies/ml and/or most recent (and within the last 6 months) CD4 count ≥ 200 cells/ml and (b) attending all scheduled clinic visits in the last 3 months assessed using the patient-held record. Stable adolescents receive a home visit once a month, plus a weekly, individualized SMS. The SMS is written and sent by the CATS and focuses on (i) motivational reminders around adherence, (ii) clinic and support group attendance and (iii) encouragement to contact the CATS in the event of any problems. An additional home visit is conducted if an adolescent misses a scheduled clinic appointment or support-group meeting
2. Adolescents in need of enhanced support are those: (a) with a viral load ≥ 1000 copies/ml and/or CD4 count < 200 cells/ml, (b) at risk of other CMDs/with a major depressive disorder (assessment methods described below), (c) failing to attend one or more scheduled clinic visits in the last 3 months, (d) commencing ART in the past 3 months, (e) who are pregnant and (f) with other psychosocial challenges or protection issues. Adolescents in need of enhanced support receive bi-weekly home visits, plus weekly phone calls and daily SMS. During the home visit, the CATS conduct an adherence assessment and provide adherence counseling as appropriate. A community health nurse accompanies the CATS where possible. If adolescents require more than one home visit every 2 weeks (for child protection cases, depression, ill-health), these are conducted by CATS with the caregiver present

Adolescents are allocated to the appropriate intervention level at enrollment, and re-assessed at 3-monthly intervals by the CATS in conjunction with the clinic nurses. Adolescents are moved between levels of intervention support as indicated by clinic attendance and other clinical factors as outlined in Table 1.

**Table 1:** Intervention components and levels of intervention support

Level of care	Standard Zvandiri support	Enhanced Zvandiri support
Eligibility criteria	• A viral load < 1000 copies/ml in last 6 months • CD4 count ≥ 200 cells/ml in last 6 months • Attending all scheduled clinic visits in last 3 months • Psychologically stable • Safe	• Commencing ART in past 3 months • A viral load ≥ 1000 copies/ml in the last 6 months • CD4 count < 200 cells/ml last 6 months • Pregnancy • Failing to attend ≥ 1 scheduled clinic visit(s) in last 3 months • Psychological distress • Abuse or neglect
Intervention	• Monthly support group • Monthly home visit • Weekly SMS reminder • Zvandiri Centre contact • Caregiver workshop	• Monthly support group • Bi-weekly home visits • Daily SMS reminder • Weekly phone calls • Zvandiri Centre contact • Caregiver workshop • Referral and linkages • Community outreach visits

*ART* antiretroviral therapy, *SMS* short message service

All participants at Zvandiri intervention sites are invited to attend a monthly support group, facilitated by a support-group leader (a volunteer nurse, teacher or social worker) in conjunction with the CATS, with supervision from the intervention coordinator. A standardized curriculum is used, focusing on improving health and treatment literacy, HIV disclosure, resilience and coping strategies, sexual and reproductive health, social networks and awareness of, and linkages to, services as required. Adolescents are actively followed up by the CATS to maximize attendance at this support group. Adolescents identified to be at risk of harm are immediately referred to the Zvandiri intervention coordinator or clinic nurse for mental health services and/or management with the Department of Social Services.

### Zvandiri intervention conceptual framework

**Fig. 2:**
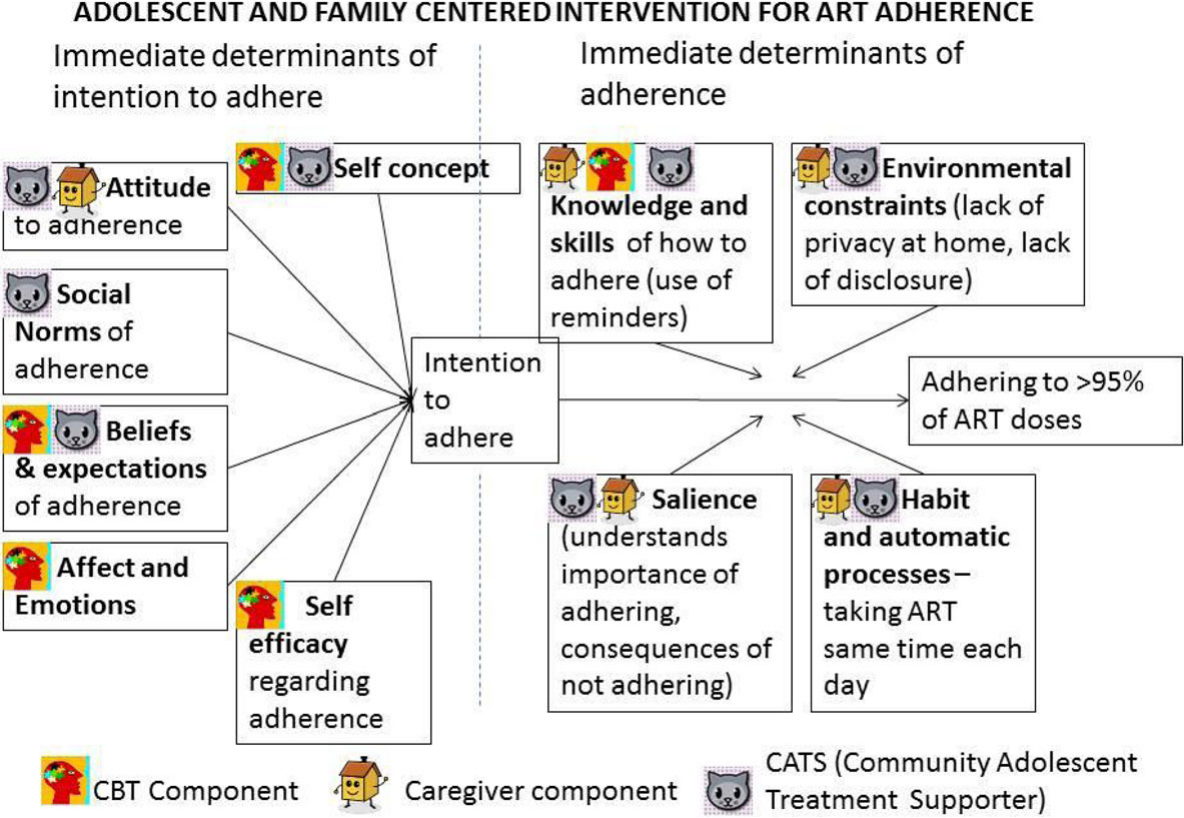
Adolescent and family centered intervention for antiretroviral therapy (ART) adherence

The Zvandiri intervention is informed by the Unified Theory of Behavior (UTB) [22, 23]. The UTB is a validated conceptual framework that has previously been adapted to support adolescent behavior change within the context of a family centered approach. The UTB conceptualizes behavior in terms of two dimensions – the immediate determinants of behavior, and of behavioral intention. It states that adherence to ART (for example) is more likely to occur if both (1) the determinants of adhering to ART and (2) the intention to adhere to ART are “aligned in favor of its enactment” [22, 23]. The five immediate determinants of behavior (i.e., ART adherence) are shown in Fig. 2, as are the immediate determinants of behavioral intention (i.e., intention to adhere to ART). We used this framework, combined with evidence from the academic literature about the determinants of adolescent HIV adherence and data from our formative work, to develop a modular Zvandiri intervention ensuring that each component of the framework is adequately addressed.

Caregivers of adolescents in the intervention arm are invited to a 12-session caregiver support group, facilitated by the intervention coordinator and CATS over 12 months. Sessions are in Shona, the participants’ language. The sessions focus on improving caregivers’ knowledge, skills and confidence of HIV and treatment literacy, communication and parenting and available support services. Two sessions are held jointly with adolescents.

### Definition of endline

The primary endpoint will be at 96 weeks post enrollment. There will be an 8-week window period on either side of this endpoint to allow flexibility in timing of visit (i.e., 88–104 weeks).

### Outcomes

The primary outcome is the proportion of participants who have died or who have a viral load ≥ 1000 copies/ml at 96 weeks, assessed on a dried blood-spot sample. Secondary outcomes are assessed at 48 and 96 weeks, and are the proportion of participants:

1. Who are not retained in clinic services stratified according to the WHO definition [24]. Full retention is defined as attending > 80% of scheduled clinic appointments; partial retention as attending 30–80% of scheduled appointments and non-retention as < 30% attendance at scheduled appointments) [24]. Retention in services will be measured through attendance at scheduled clinic visits (scheduled visit defined as visit within ± 7 days of exact date)
2. Who discontinue ART defined as completely stopping taking drugs for at least 3 months, as documented in clinic records (3 months is the minimum period associated with risk of progression to new clinical AIDS events or death) [25, 26]
3. With depression, defined as scoring ≥ 10/27 on the Patient Health Questionnaire (PHQ-9), a scale that has been widely used in Africa [27, 28] and recently validated for use in Zimbabwe [29]
4. With CMDs (depression and/or anxiety), defined as scoring ≥ 8/14 on the Shona Symptom Questionnaire (SSQ-14) which was developed and validated in Zimbabwe using exemplary cross-cultural methods [30]. This cut-point may be amended prior to locking the analysis plan, based on results of a separate validation study. Only two of its items relate to somatic symptoms making it useful in participants with physical disease. Severity of CMDs will also be assessed using the SSQ-14 as a continuous score
5. With poor quality of life, measured using a previously-validated Shona version of the European Quality of Life-5 Dimensions (EQ-5D) scale [31, 32]

Exploratory outcomes are assessed at 48 and 96 weeks, and are the proportion of participants:

1. Self-reporting disclosure of HIV status to sexual partners, assessed using a self-completed questionnaire
2. Self-reporting perceived stigma measured using the HIV/AIDS Stigma Instrument for people living with AIDS (PLWA) (HASI-P) which has been validated in Africa [33]

### Sample size and potential power of the trial

The total sample size of 500 participants recruited from 16 clusters will provide 80% power to detect a difference in detectable viral load of 35% among participants in the standard-care arm versus 18% in the intervention arm assuming 20% loss to follow-up and a coefficient of variation (*k*) between clusters of 0.25 [34]. For secondary outcomes, the trial will have 90% power to detect, for example, a difference in non-complete attendance of 26% in the standard-care arm versus 10% in the intervention arm, and 80% power to detect a difference in mean SSQ-14 [30] score of 7.4 in the control arm and 4.8 in the intervention arm (standard deviation (SD) = 3.74).

### Study schedule

**Fig. 3:**
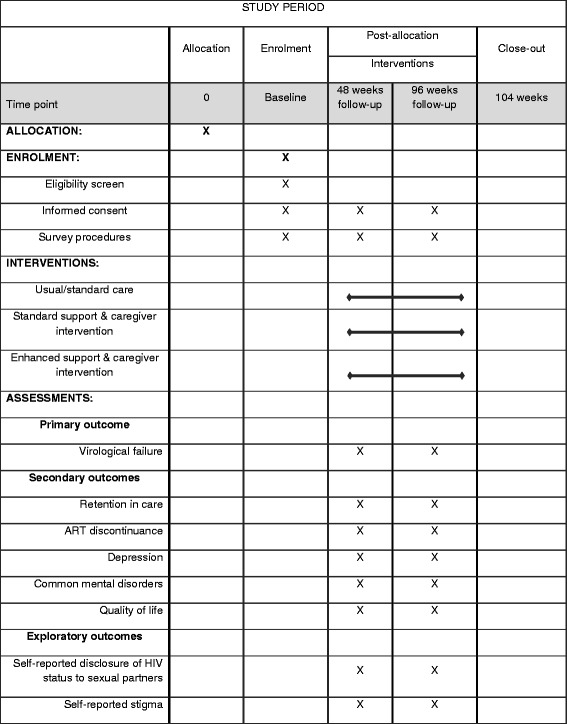
Schedule of enrollment, interventions and assessments following the Standard Protocol Items: Recommendations for Intervention Trials (SPIRIT) guidelines

Figure 3 shows the study schedule which follows the Standard Protocol Items: Recommendations for Intervention Trials (SPIRIT) guidelines.

### Randomization

Imbalance between arms was minimized by using restricted randomization and matching of clusters on size and number of people in HIV care. Sixteen clusters were allocated 1:1 to either the immediate intervention or the control arm. To maximize transparency and buy-in from key stakeholders, a public randomization procedure was undertaken on 5 July 2016 involving MoHCC, Community Advisory Board members, and district-level governance and medical representatives.

### Recruitment and enrollment procedures

From the 16 clusters, a list of eligible participants was generated from the “pre-ART and ART Registers.” At each cluster, selected village healthcare workers (VHCWs) were asked to mobilize potential participants after receiving training on trial objectives and procedures, as well as confidentiality aspects. The research team then held a trial-orientation meeting with the potential participants plus their caregivers. After orientation and screening, participants and caregivers who opted to take part in the trial and provided written consent, plus assent to enroll, were booked for enrollment procedures and baseline assessments. Recruitment and enrollment in the intervention and control arms occurred concurrently. At enrollment, participants were informed that they would be allocated to either the intervention or the control arm and told which one they were allocated to afterwards. Recruitment started on 15 August 2016 and concluded on 31 March 2017.

### Quantitative data collection and management

At enrollment, participants complete a questionnaire using an interviewer-administered, computer-assisted personal interview (CAPI) with more sensitive questions likely subject to socially desirability bias self-administered using an audio-computer-assisted survey instrument (ACASI) [35, 36]. Questionnaire domains include: sociodemographic, socioeconomic, medical history, including history of opportunistic infections and hospitalization, HIV-testing history, ART history, information on clinic attendances, information on adherence (self-report), and psychological wellbeing. On completion of the questionnaire, all participants are asked to provide a finger-prick dried blood-spot sample for HIV viral-load testing and undergo a clinical exam to assess WHO stage. All viral-load test results will be returned to the clinic within 3 months to guide clinical care (in both the intervention and comparison arms). All trial participants are assessed by the research team, in addition to their routine clinic visits, after 48 and 96 weeks. At the follow-up visit, they will complete a shortened version of the questionnaire, have a finger-prick blood-spot sample taken for viral-load testing and have a physical examination to allow clinical staging.

#### Quantitative data analysis

Data collected using ACASI and CAPI is downloaded into a password-protected Access database. The data will be analyzed using Stata 14. Statistical analyses will be conducted and reported in line with Consolidated Standards of Reporting Trials (CONSORT) guidelines [37] including a flowchart. Baseline characteristics of enrolled participants and the number continuing through the trial, actively withdrawing, and passively lost to follow-up will be shown by arm. The outcome measures will be summarized at baseline, 48 and 96 weeks by intervention arm, using proportions or means (SD) as appropriate.

Analyses will be intention-to-treat. Analyses will be based on cluster-level summary measures, as individual-level regression methods do not perform robustly when there are relatively few clusters per arm, especially for stratified cluster randomized trials [38]. For binary outcomes, the impact will be estimated by the risk ratio (RR) and risk difference (RD). The stratum-specific risk ratios will be estimated as the ratio of the geometric mean risks between arms for each of the two strata, and the overall RR will be estimated as the weighted-average of these stratum-specific risk ratios. An approximate variance for the log of the mean risk in each arm will be estimated from the residual mean square from a two-way analysis of variance of community log-risk on strata and study arm. A 95% confidence interval (CI) for the RR will be estimated from this variance using a stratified *t* test with 14 degrees of freedom. Similarly, a 95% CI for the RD will be estimated from an analysis of variance of the mean risk on strata and study arm. For continuous outcomes, the measure of effect will be the mean difference between arms, analyzed in an analogous method based on mean scores in each facility.

Secondary planned analyses will examine the impact of the intervention separately by age group, gender, duration on treatment, with assessment of effect-modification of the intervention effect by facility type using a previously described method [39]. Pre-defined sensitivity analyses will include adjustment for baseline viral load and other variables imbalanced at baseline. We will conduct sensitivity analyses to investigate the effect of missing data by using multiple imputation, analyzed using an individual-level Poisson regression model, allowing for within-cluster correlation using generalized estimating equations.

### Qualitative data collection and management

Twenty-six participants (20 adolescents – 10 male; 10 female – from across the age range plus six carers) from two intervention and two control communities will be purposively selected for three serial, in-depth interviews to understand the experiences of clinical care for children and young people living with HIV. In addition, four focus group discussions will be conducted with purposively selected participants and healthcare workers in both the enhanced intervention- and standard-care arms. All discussions will be audio-recorded; hand-written notes will be taken down as back-up.

#### Qualitative data analysis

Audio-recordings will be transcribed, translated into English, and coded by two independent coders in NVivo (QSR International, Melbourne, VIC, Australia). Coders will discuss discrepancies until consensus is reached. Codes will be grouped and emerging themes will then be identified. Analytic memos will be written for each theme. During write-up, themes and sub-themes will be illustrated with verbatim quotes.

### Cost-effectiveness data collection and analysis

A cost-effectiveness analysis will be conducted using standardized survey instruments and taking a provider perspective, combining top-down and bottom-up costing. Study instruments will assemble data from program records and facility staff on relative costs of personnel, recurrent inputs and services, and capital costs (equipment, vehicles and training) which will be discounted at a 3% rate. Any donated inputs will be valued at their opportunity costs determined using local market prices. Training and supervision will be valued according to opportunity cost of staff and CATS’ time dedicated to these activities. Annual staff (providers and CATS) costs will be derived from staff roster information from program records and self-reports at the facility level; grades and salaries for each type of staff; and time and motion observation (the “gold standard” in measuring staff allocation of time through direct observation).

All cost data will be collected prospectively by month and analyzed in US dollars (the US$ has been the principal currency in Zimbabwe since 2009). Any parameter uncertainty, such as influence of staff time per client, will be dealt with using univariate or multi-variate sensitivity analysis. As retention-in-care data (from trial) is only an intermediary measure of impact, we will use appropriate health-related quality of life (QALY) measures (e.g., EQ-5D) to estimate QALYs gained for the purposes of cost-effectiveness analysis. As intervention participants may transition between different states (standard Zvandiri support or enhanced Zvandiri support) over the course of the trial, a dynamic Markov model [40] will be used to track costs of care and utility at each point in time. A more realistic incremental cost-effectiveness ratio (ICER) threshold, WHO-CHOICE, which recommends a country gross domestic product (GDP)-dependent approach, will be adopted (http://www.who.int/choice).

### Process evaluation data collection and management

**Fig. 4:**
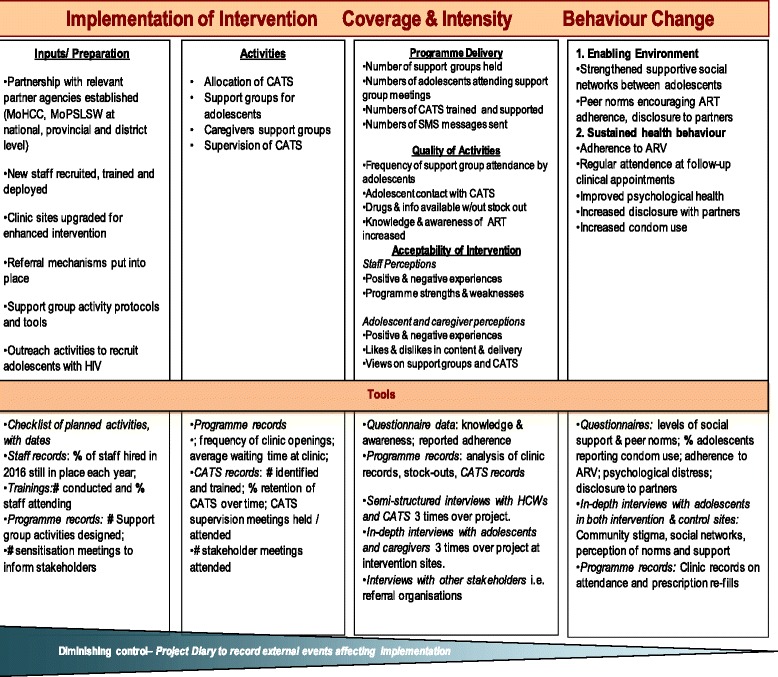
Intervention Monitoring and Process Evaluation framework

The Intervention Monitoring and Process Evaluation framework provides a structure for data collection to capture progress along the hypothesized pathway from intervention activities to their intended outcomes. For the process evaluation, we hypothesize that:

- If there are adequate inputs (human resources, partnerships and collaboration, established procedures and protocols, effective trainings)
- These will ensure a smooth process of implementation,
- producing well-functioning outputs that will contribute to the achievement of the intervention’s ultimate goals (improved physical and psychological health) (see Fig. 4)

#### Program records

Program data will be collected in intervention communities and regularly compiled to record uptake and attendance at community intervention, visits by CATS and use of clinical services.

#### Process evaluation data analysis

Service statistics and other quantitative data will be double entered into a password-protected Access database. Range and consistency checks will be performed. Quantitative data will be analyzed using Stata 14. Process evaluation qualitative data will be analyzed in the same manner as other qualitative data (already described).

## Discussion

Effective strategies are urgently needed to reach the ambitious UNAIDS 90-90-90 target by 2020 (that 90% of all people living with HIV will know their HIV status, 90% of all people with diagnosed HIV infection are receiving sustained ART, and 90% of all people receiving ART are virologically suppressed) [41]. To achieve this, high-quality programs will need to ensure high levels of retention and adherence [11, 12].

Evidence-based strategies to support both adherence and retention in care of young people are required. This trial has the potential to generate robust evidence of the impact of the Zvandiri program as delivered in a real-world setting. If found to reduce rates of treatment failure, this would strengthen support for further scale-up across Zimbabwe and likely the region more widely. In addition, the results will directly influence policies and standard of care for HIV-infected children in Zimbabwe, regionally and internationally.

While the overall trial will answer the primary research question, “*Does the Zvandiri program improve the physical and psychological health of adolescents living with HIV resulting in improved clinical outcomes and quality of life?*” process evaluation and qualitative research will provide some understanding of *why* and *how* the intervention was able to lead to improved retention/adherence along the HIV-care continuum (or suggest reasons for *lack* of effect). Additionally, process evaluation and qualitative research will suggest how the Zvandiri intervention could be modified for optimal impact during any further scale-up.

Finally, most countries including Zimbabwe have revised their HIV treatment guidelines in line with the WHO 2015 revised treatment guidelines which recommend a “test-and-start approach” for all those who are HIV infected, not just pregnant women [17]. Since a significant proportion of the likely beneficiaries of “test and start” are likely to be young people, this trial will help identify some of the challenges of this approach. Specifically, the trial will provide needed information on the capacity of primary care facilities to provide effective adherence support as well as support for the broader psychosocial issues that many of these young people face.

### Trial status

At the time of initial manuscript submission recruitment for this trial was on-going but not yet complete.

## Abbreviations

ACASI: Audio-computer-assisted survey instrument
AIDS: Acquired immunodeficiency syndrome
ART: Antiretroviral therapy
CAPI: Computer-assisted personal interview
CATS: Community adolescent treatment supporters
CBT: Cognitive behavioral therapy
CD4: Cluster of differentiation 4
CI: Confidence interval
CONSORT: Consolidated Standards of Reporting Trials
cRCT: Cluster randomized controlled trial
DSMB: Data Safety and Monitoring Board
EQ-5D: European Quality of Life-5 Dimensions
GDP: Gross domestic product
HASI-P: HIV/AIDS Stigma Instrument – PLWA
HIV: Human immunodeficiency virus
ICER: Incremental cost-effectiveness ratio
LMIC: Low- and middle-income countries
MoHCC: Ministry of Health and Child Care (Zimbabwe)
PHQ: Patient Health Questionnaire
PLWA: People living with AIDS
QALY: Health-related quality of life
RD: Risk difference
RR: Risk ratio
SD: Standard deviation
SMS: Short message service
SPIRIT: Standard Protocol Items: Recommendations for Intervention Trials
SSQ: Shona Symptom Questionnaire
UNAIDS: Joint United Nations Program for HIV
USAID: United States Agency for International Development
UTB: Unified Theory of Behavior
VHCW: Village healthcare worker
WHO: World Health Organization
